# IκBα kinase inhibitor BAY 11-7082 promotes anti-tumor effect in RAS-driven cancers

**DOI:** 10.1186/s12967-024-05384-4

**Published:** 2024-07-09

**Authors:** Praveen Guruvaiah, Romi Gupta

**Affiliations:** 1 Department of Biochemistry and Molecular Genetics, The University of Alabama at Birmingham, Birmingham, AL 35233 USA; 2 O’Neal Comprehensive Cancer Center, The University of Alabama at Birmingham, Birmingham, AL 35233 USA

**Keywords:** NRAS, KRAS, HRAS, MAPK, PI3K-AKT

## Abstract

**Background:**

Oncogenic mutations in the RAS gene are associated with uncontrolled cell growth, a hallmark feature contributing to tumorigenesis. While diverse therapeutic strategies have been diligently applied to treat RAS-mutant cancers, successful targeting of the RAS gene remains a persistent challenge in the field of cancer therapy. In our study, we discover a promising avenue for addressing this challenge.

**Methods:**

In this study, we tested the viability of several cell lines carrying oncogenic NRAS, KRAS, and HRAS mutations upon treatment with IkappaBalpha (IκBα) inhibitor BAY 11-7082. We performed both cell culture-based viability assay and in vivo subcutaneous xenograft-based assay to confirm the growth inhibitory effect of BAY 11-7082. We also performed large RNA sequencing analysis to identify differentially regulated genes and pathways in the context of oncogenic NRAS, KRAS, and HRAS mutations upon treatment with BAY 11-7082.

**Results:**

We demonstrate that oncogenic NRAS, KRAS, and HRAS activate the expression of IκBα kinase. BAY 11-7082, an inhibitor of IκBα kinase, attenuates the growth of NRAS, KRAS, and HRAS mutant cancer cells in cell culture and in mouse model. Mechanistically, BAY 11-7082 inhibitor treatment leads to suppression of the PI3K-AKT signaling pathway and activation of apoptosis in all RAS mutant cell lines. Additionally, we find that BAY 11-7082 treatment results in the downregulation of different biological pathways depending upon the type of RAS protein that may also contribute to tumor growth inhibition.

**Conclusion:**

Our study identifies BAY 11-7082 to be an efficacious inhibitor for treating RAS oncogene (HRAS, KRAS, and NRAS) mutant cancer cells. This finding provides new therapeutic opportunity for effective treatment of RAS-mutant cancers.

**Supplementary Information:**

The online version contains supplementary material available at 10.1186/s12967-024-05384-4.

## Introduction

The Rat sarcoma (RAS) family consists of several genes that encode proteins involved cell signaling pathways and key cellular processes such as cell proliferation, differentiation, and survival [1]. The three main RAS genes that are frequently mutated in human cancers include Harvey rat sarcoma viral oncogene homolog (HRAS), Kirsten rat sarcoma viral oncogene homolog (KRAS), and Neuroblastoma ras viral oncogene homolog (NRAS). Oncogenic mutations in these genes activate signaling pathways that promote uncontrolled cell growth and contribute to tumorigenesis [1]. Notably, KRAS mutations (G12, G13 or Q61) are often identified in various cancers such as lung, pancreatic, colorectal, and certain types of leukemia [2]. Oncogenic KRAS mutations regulates several aspects of tumor growth and development including promoting tumor growth, metastasis, angiogenesis, resisting apoptosis, promoting immune evasion and also contributing to resistance to therapies [2, 3]. Consequently, understanding KRAS biology has become a focal point in developing targeted therapies and improving treatment outcomes for KRAS-related cancers. For a long time, KRAS was considered “undruggable,” but recent studies have identified therapies targeting specific KRAS mutations, especially in treating predominantly RAS-driven cancers [4].

Similarly, oncogenic mutations in the NRAS gene at codon 12, 13 and 61 are observed in various cancers including melanoma, colorectal cancer, and certain types of leukemia [5, 6]. Notably, melanoma exhibits a relatively high frequency of NRAS mutations [7]. Oncogenic NRAS mutations have shown to regulate cancer cell proliferation, metastasis, and response to targeted therapies and chemotherapy by modulating several oncogenic signaling pathways such as MAPK pathway, PI3K-AKT signaling pathway among others [7, 8]. They have also been shown to regulate epigenetic changes in the cancer cells [9]. Targeting NRAS mutant cancers is challenging and treatment strategies include targeting NRAS-regulated mitogen activated protein kinase (MAPK)/extracellular signal-regulated kinase (ERK) signaling pathways either alone or in combination with PI3K inhibitors or immunotherapies [10, 11].

HRAS mutations, on the other hand, are less common and are associated with dermatological cancer and head and neck squamous cell carcinoma [12]. Oncogenic HRAS mutations are also shown to regulate several intracellular signaling pathways like MAPK, PI3K-AKT to enhance cell proliferation and resist apoptosis [13, 14]. Just like NRAS, targeting HRAS mutations has been challenging, and the treatment involves mitogen-activated protein kinase kinase (MEK) or phosphoinositide 3-kinase (PI3K) inhibitors [15].

Targeting the RAS gene has been a longstanding challenge in the area of cancer therapy [16]. This has been attributed to its highly mutated nature, its unique structure configuration lacking well-defined pockets for small molecule binding, multiple isoforms exhibiting distinct functions across various cell types, and a propensity for developing resistance to existing inhibitors over time [17, 18]. Thus, there is a need to explore different approaches for developing effective RAS-targeted therapies and discovering efficacious inhibitors that can effectively target all RAS forms.

Our goal in the current study is to discover an inhibitor that is capable of targeting all RAS protein family members, NRAS, KRAS and HRAS efficiently. We find that oncogenic NRAS, KRAS, and HRAS activate the expression of IkappaBalpha (IκBα) and BAY 11-7082, an inhibitor of IκBα kinase, attenuates the growth of NRAS, KRAS, and HRAS mutant cancer cells in cell culture and in vivo mouse model. BAY 11-7082 inhibitor treatment leads to suppression of the PI3K-AKT signaling pathway and activation of apoptosis in all RAS mutant cell lines. Additionally, BAY 11-7082 inhibitor treatment leads to downregulation of several specific oncogenic signaling pathways in NRAS, KRAS, and HRAS mutant cancer cells. These findings suggest that BAY 11-7082 holds significant promise as an anti-cancer compound, with potential therapeutic applications across a spectrum of RAS-driven cancers.

## Results

### Identification and targeting of IκBα kinase in NRAS, KRAS, and HRAS mutant cancer cells

In our previous study, in order to identify the genes driven by oncogenic variants of NRAS, KRAS, and HRAS, we overexpressed different mutant oncogenic RAS (HRASV12, KRASV12, and NRASQ61K) in immortalized melanocytes (MEL-ST) cells. As a control, MEL-ST cells transfected with vectors control was used. Transcriptome wide gene expression analyses were performed using the Illumina HumanHT-12 V4.0 Expression BeadChip array (GEO accession number: GSE62827) [19] and the common genes that were significantly altered in HRASV12, KRASV12, and NRASQ61K overexpressing cells as compared with the control vector expressing MEL-ST cells were identified. This analysis revealed that oncogenic forms of NRAS, KRAS, and HRAS (HRASV12, KRAS V12, and NRASQ61K) activate the expression of several genes including IkappaBalpha (IκBα) kinase. IκBα, a member of the NF-kappa-B (NF-κB) inhibitor family characterized by multiple ankrin repeat domains, is pivotal in regulating the NF-κB signaling pathway [20]. This pathway plays a central role in regulating immune and inflammatory responses and cell survival, proliferation, and differentiation [20].

**Fig. 1:**
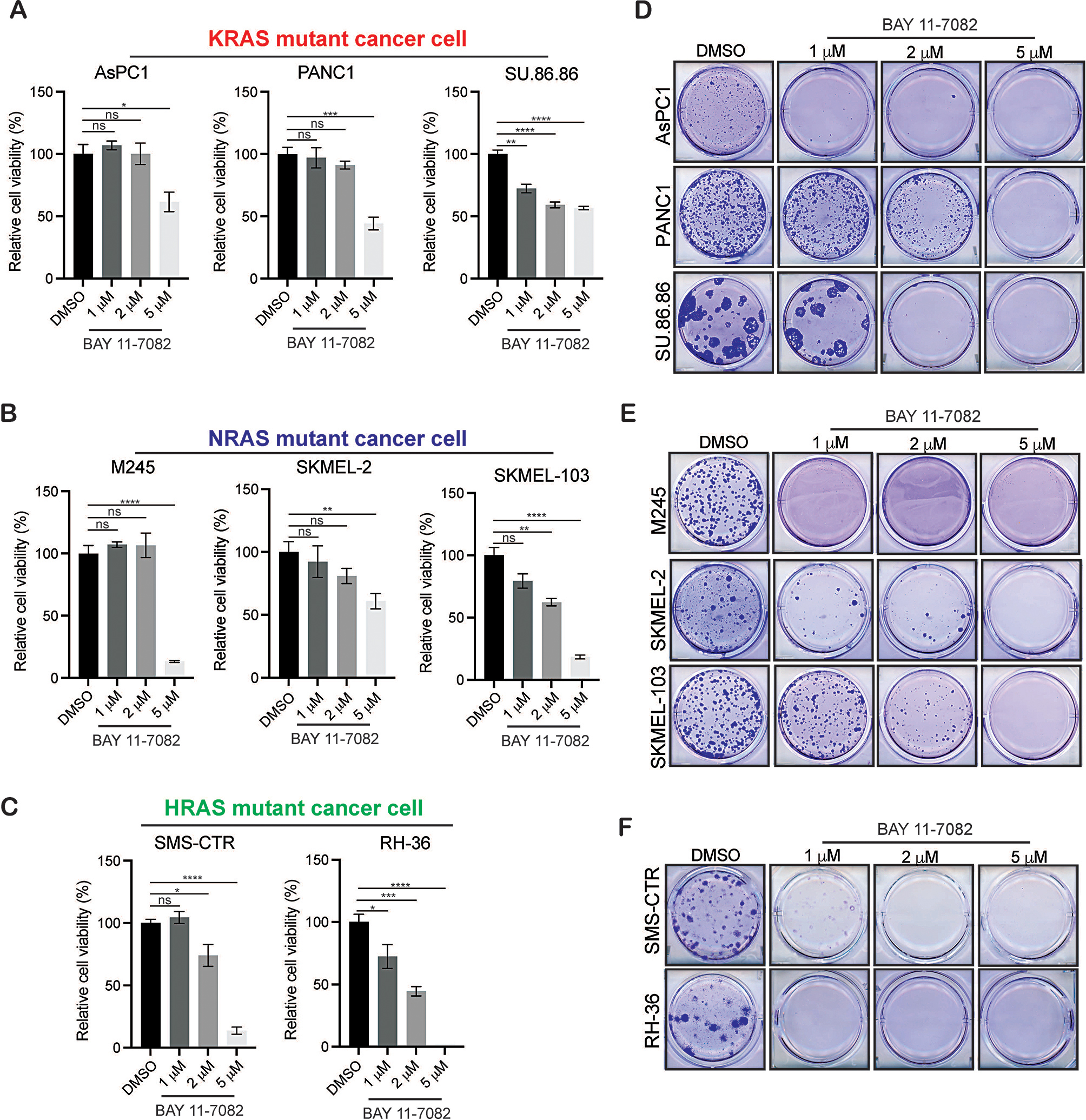
BAY 11-7082 treatment inhibits the growth of NRAS, KRAS, and HRAS mutant cancer cells. **A**-**C**. The indicated cancer cell lines were treated with various concentration of BAY 11-7082 for three days and subjected to MTT assays. Relative percentage cell viability was plotted with respect to DMSO treated cells. **D-F.** The indicated cancer cell lines were treated with various concentration of BAY 11-7082 for 2–4 weeks, and long-term cell survival was measured using clonogenic assays. Representative images are shown. Data represent the mean ± standard error of three biological replicates. ns = not significant, *p < 0.05, **p < 0.01, ***p < 0.001, ****p < 0.0001

To understand IκBα kinase’s role in regulating RAS-mediated tumorigenesis, we employed BAY 11-7082, a broad-spectrum IκBα kinase inhibitor with potential therapeutic value in various inflammation-driven diseases [21]. We treated multiple NRAS (SKMEL-103, M245, and SKMEL-2), KRAS (AsPC1, PANC1, and SU.86.86), and HRAS (RH-36 and SMS-CTR) mutant cancer cell lines with different concentrations of BAY 11-7082 and measured cell viability. Using different concentrations of a BAY 11-7082 and varying the duration of treatment is important for finding the optimal dose with the maximum tumor inhibitory effect. We performed short-term survival using a 3-(4,5-dimethylthiazol-2-yl)-2,5-diphenyl-2 H-tetrazolium bromide (MTT) assay and long-term survival using a clonogenic assay. In both MTT and clonogenic assay, we observed that the treatment of cancer cells expressing different oncogenic RAS with BAY 11-7082 led to inhibition of short-term (Fig. 1A–C) and long-term survival (Fig. 1D–F) of cells in a concentration-dependent manner.

**Fig. 2:**
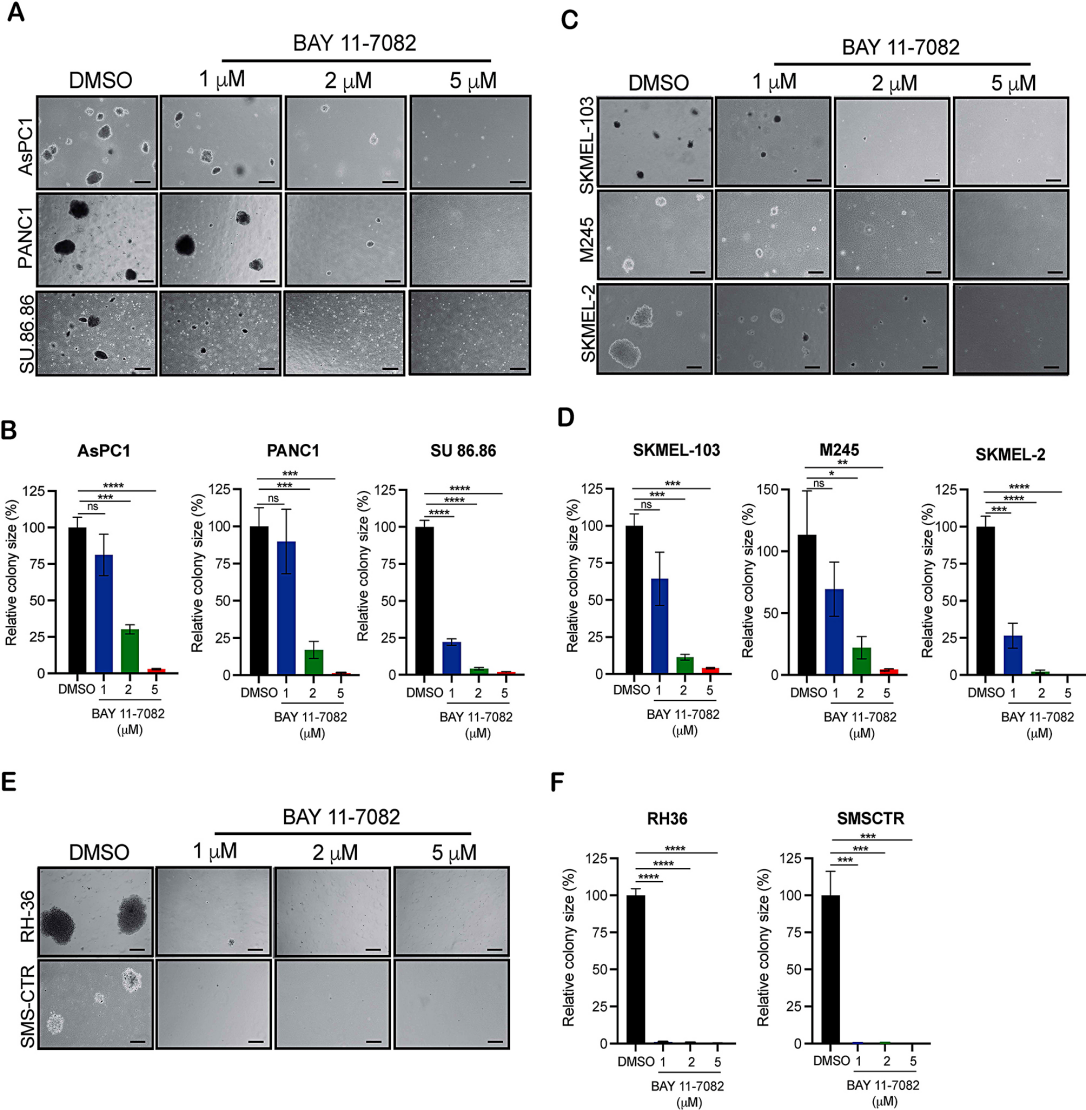
BAY 11-7082 treatment inhibits the tumor growth of NRAS, KRAS, and HRAS mutant cancer cells in vitro. **A**, **C**, **E**. The indicated cancer cell lines were treated with different concentrations of BAY 11-7082 and analyzed for their ability to grow in soft agar. Representative images are shown; scale bar, 500 μm. **B, D, F.** Relative colony size for the images shown in panels A, C and E respectively. Data represent the mean ± standard error of three biological replicates. ns = not significant, *p < 0.05, **p < 0.01, ****p < 0.001 and ****p < 0.0001

We subsequently evaluated BAY 11-7082’s efficacy in inhibiting the tumorigenic potential of cancer cells expressing oncogenic NRAS, KRAS, and HRAS mutations. We first performed soft agar assay, a surrogate assay to measure the tumorigenic potential of cancer cells [22]. Results indicated a significant, concentration-dependent reduction in tumor-forming potential of cancer cells expressing oncogenic NRAS, KRAS, and HRAS mutations upon treatment with BAY 11-7092 (Fig. 2A–F).

**Fig. 3:**
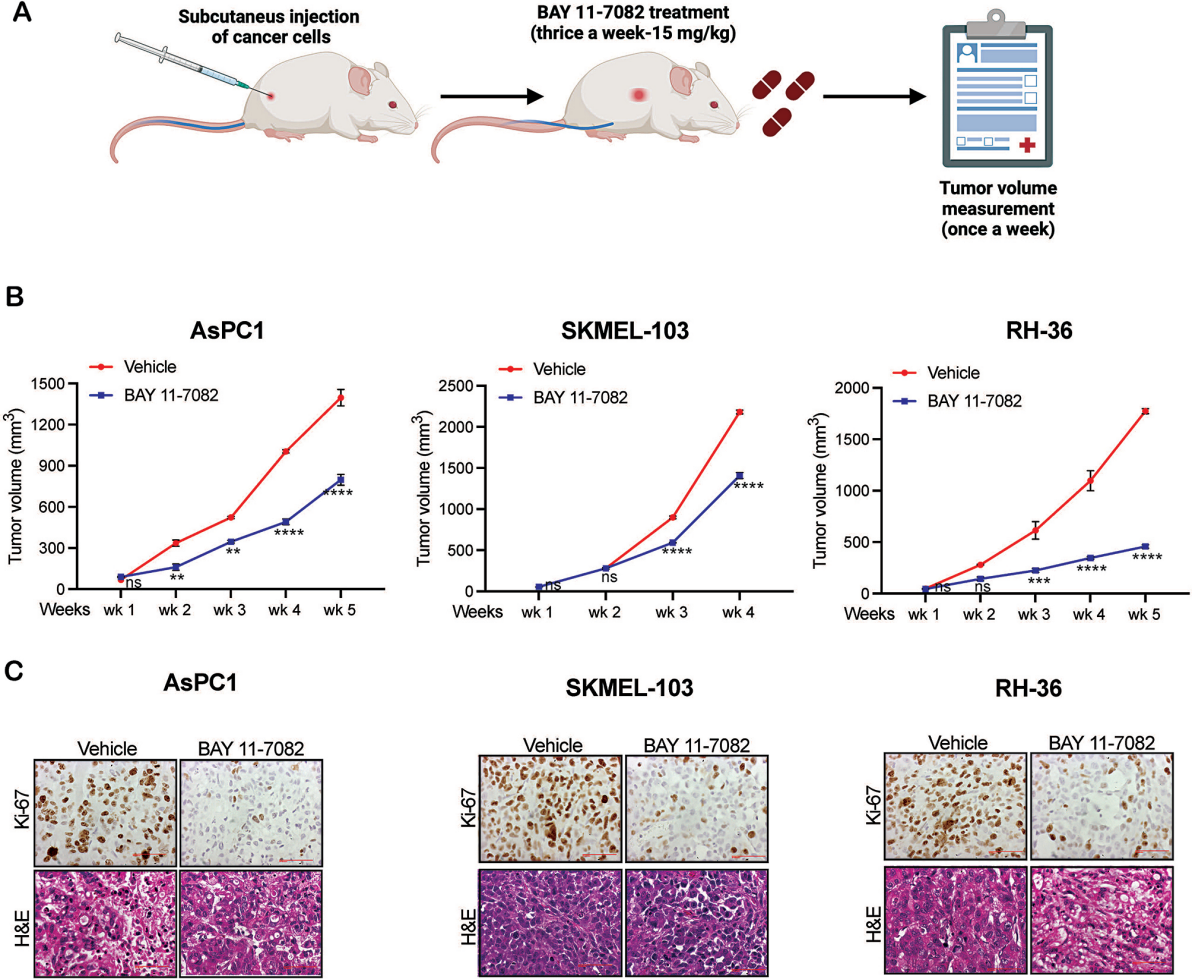
BAY 11-7082 treatment inhibits tumor growth of NRAS, KRAS, and HRAS mutant cancer cells in vivo. **(A)** Schematics of performing the assay. **(B)** Indicated cancer cell lines were subcutaneously injected into the flanks of NSG mice (*n* = 6). The mice were treated with vehicle or BAY 11-7082 (15 mg/kg body weight) intraperitoneally every other day. The average tumor volumes were plotted each week. **(C)** Tumors from the vehicle or BAY 11-7082 treated mice were harvested at the endpoint, as shown in Figure B, sectioned and stained for H&E and Ki-67. Representative images are shown. Data represent the mean ± standard error of three biological replicates. ***p < 0.001

Based on in vitro based soft agar results, the effect of BAY 11-7082 was investigated in in vivo subcutaneous xenograft-based mouse model. NRAS-mutant SKMEL-103 cell, KRAS- mutant AsPC1 cells, and HRAS-mutant RH-36 cells were injected into the flanks of immunodeficient NSG mice. These mice were treated with either vehicle (30% PEG-300 and 5% tween 80) or BAY 11-7082 (15 mg/kg), and tumor growth was monitored weekly (Fig. 3A). We observed that BAY 11-7082 markedly suppressed the growth of SKMEL-103, AsPC1, and RH-36 cell-derived tumors (Fig. 3B). Histological examination of the tumor tissues, stained with Hematoxylin and Eosin (H&E) and Ki-67 (a marker of tumor proliferation), revealed a reduction in Ki-67 positive cells in BAY 11-7082-treated tumors compared to the control vehicle treated group (Fig. 3C). Additionally, examination of tumor tissue for cleaved caspase 3 (a marker for apoptosis) in BAY 11-7082-treated tumors revealed higher cleaved caspase 3 level as compared to control vehicle treated tumors (Supplementary Fig. 1). These results demonstrated that BAY 11-7082 effectively inhibits the growth of multiple cancer cell lines expressing different oncogenic RAS such as NRAS, KRAS, and HRAS in both in vitro and in vivo assays.

### IκBα kinase inhibitor promotes apoptosis in NRAS, KRAS, and HRAS mutant cancer cells

**Fig. 4:**
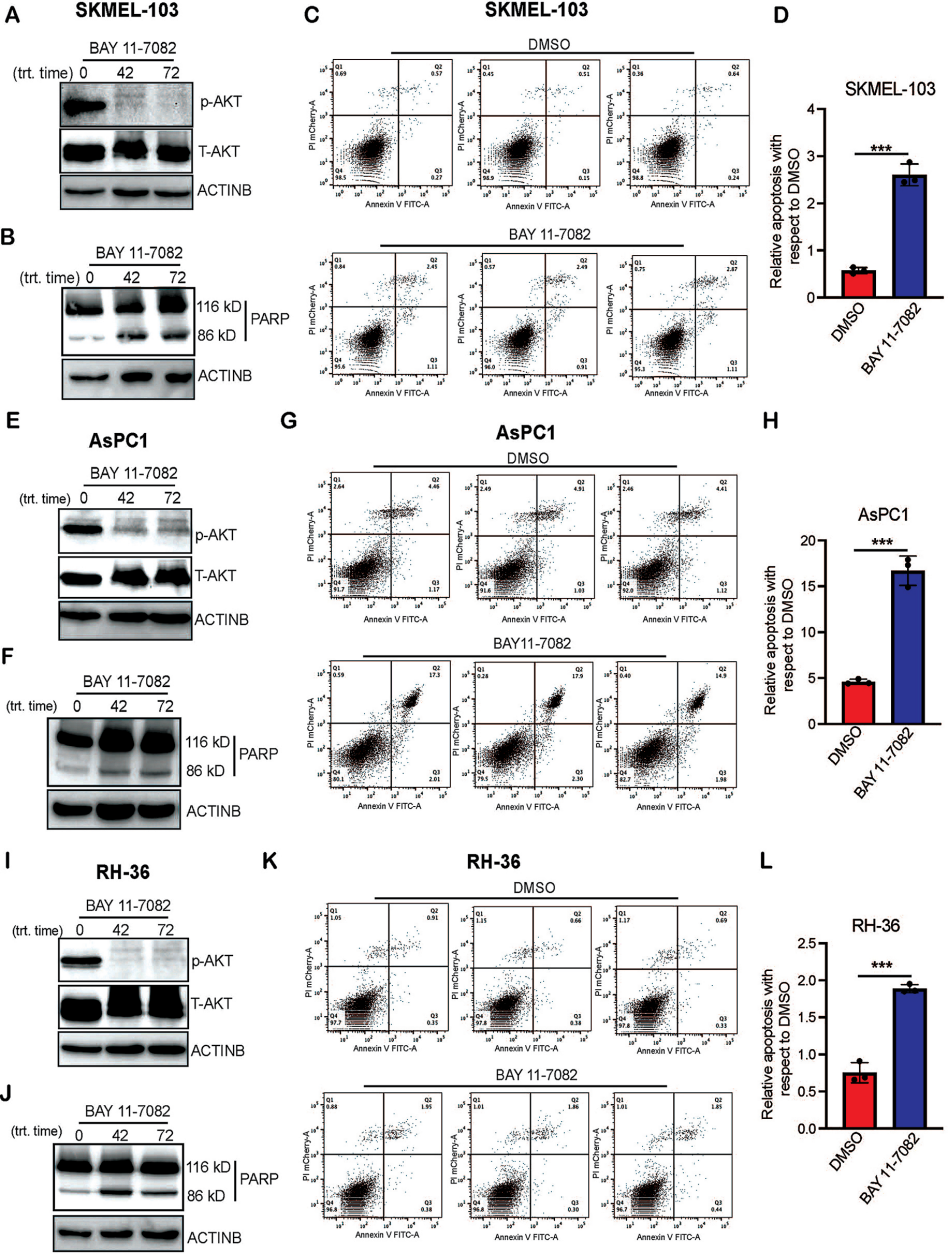
BAY 11-7082 treatment inhibits PI3K-AKT signaling and induces apoptosis in NRAS, KRAS, and HRAS mutant cancer cells. **A**, **E**, **I**. The indicated cancer cell lines were treated with BAY 11-7082 (5 µM) for 48 and 72 h, and phosphorylated AKT and total AKT were measured via immunoblotting. ACTINB was used as a loading control. **B**, **F**, **J**. The indicated cancer cell lines were treated with BAY 11-7082 (5 µM) for 48 and 72 h. Cleaved PARP was measured using immunoblotting. ACTINB was used as a loading control. **C**, **G**, **K**. The indicated cancer cell lines were treated with BAY 11-7082 (5 µM) for 48 h, and apoptosis was measured using an annexin V/propidium iodide staining kit. **D**, **H**, **L**. The Q2 population from the shown data in panels C, G, and K is plotted in DMSO or BAY 11-7082 treated cells. Data represent the mean ± standard error of three biological replicates. **p < 0.01, ****p < 0.0001

BAY 11-7082 is known to suppress prooncogenic signaling pathways, induce apoptosis, and inhibit the proliferation of cancer cells [21]. Therefore, we first analyzed the effect of BAY 11-7082 on MAP kinase and PI3K-AKT signaling pathways because mutant RAS proteins have shown to mediate its oncogenic effect by activating the MAP kinase and PI3K-AKT signaling pathways [23, 24]. We found that PI3K-AKT signaling pathway was significantly inhibited in NRAS, KRAS, and HRAS mutant-cancer cell lines upon treatment with BAY 11-7082 as observed by reduced p-AKT protein level (Fig. 4A, E, I). However, we did not observe any significant reduction in MAPK signaling pathway following BAY 11-7082 treatment (Supplementary Fig. 2). These results demonstrate that BAY 11-7082 primarily suppresses the PI3K-AKT signaling pathways in RAS-driven cell lines. We next measured the apoptosis induction using annexin V and PI staining method and found a significant increase in apoptosis induction in NRAS, KRAS, and HRAS mutant-cancer cell lines upon treatment with BAY 11-7082 as compared to DMSO treated cells (Fig. 4C–D, G–H, K–L). Further validation on increases apoptosis upon treatment with BAY 11-7082 was performed using PARP-cleavage assay. We observed similar results, namely BAY 11-7082 treatment leads to significant increase in PARP-cleavage in NRAS, KRAS, and HRAS mutant-cancer cell lines (Fig. 4B, F, J). These results confirmed that BAY 11-7082 effectively suppresses PI3K-AKT signaling and induces apoptosis in all RAS mutant cell lines.

### IκBα kinase inhibitor treatment in NRAS, KRAS, and HRAS mutant cancer cells changes specific signaling pathways that regulate tumor growth

**Fig. 5:**
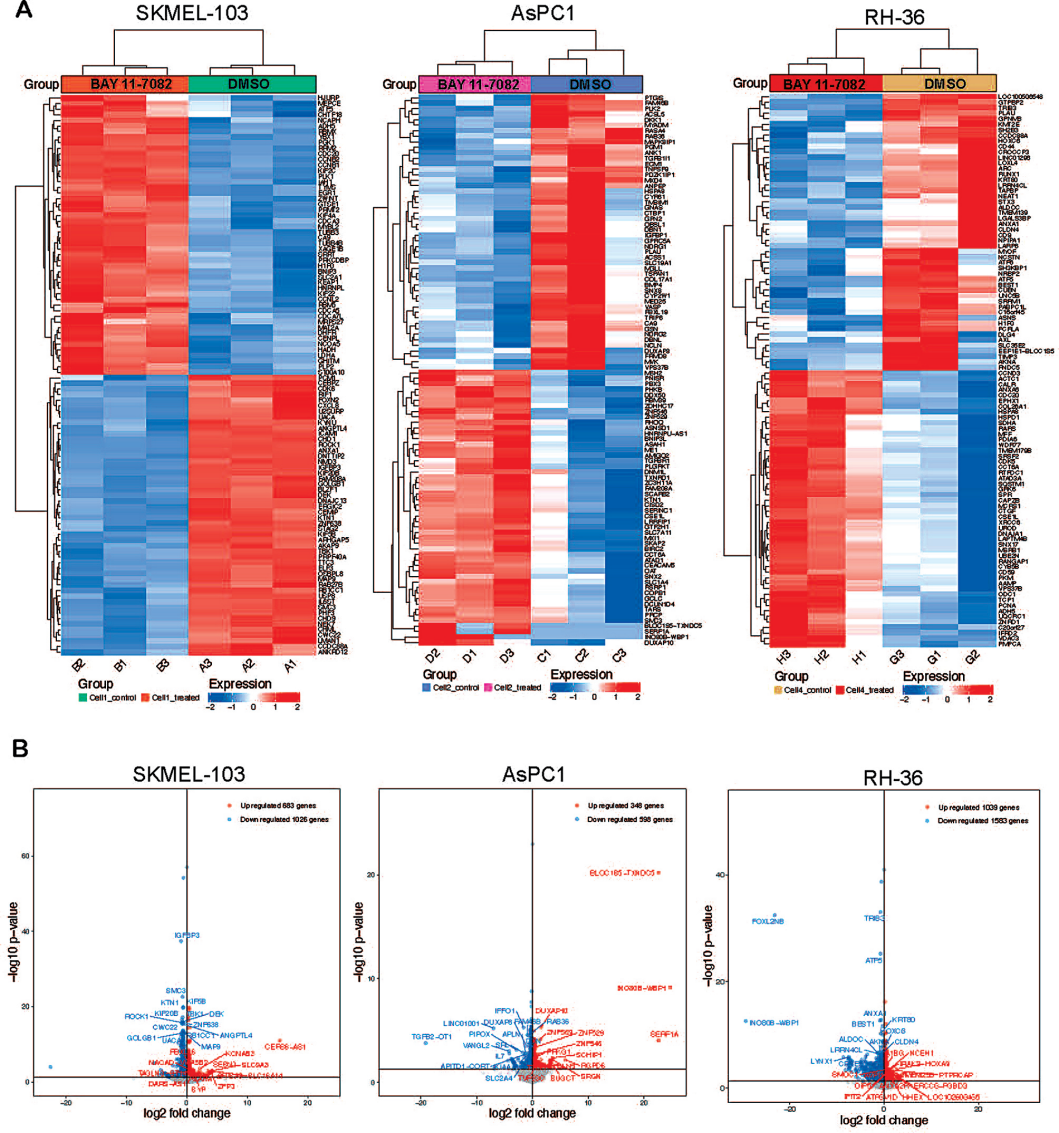
BAY 11-7082 treatment alters the expression of multiple genes in NRAS, KRAS, and HRAS mutant cancer cells. **(A)** Heatmaps showing the top 50 upregulated or downregulated genes in SKMEL-103, AsPC1, and RH-36 cells upon treatment with BAY 11-7082 (5 µM) for 48 h as compared with DMSO-treated cells. **(B)** Volcano plot showing top 15 genes upregulated or downregulated after 48-h treatment with BAY 11-7082 (5 µM) in RAS mutant cells

**Fig. 6:**
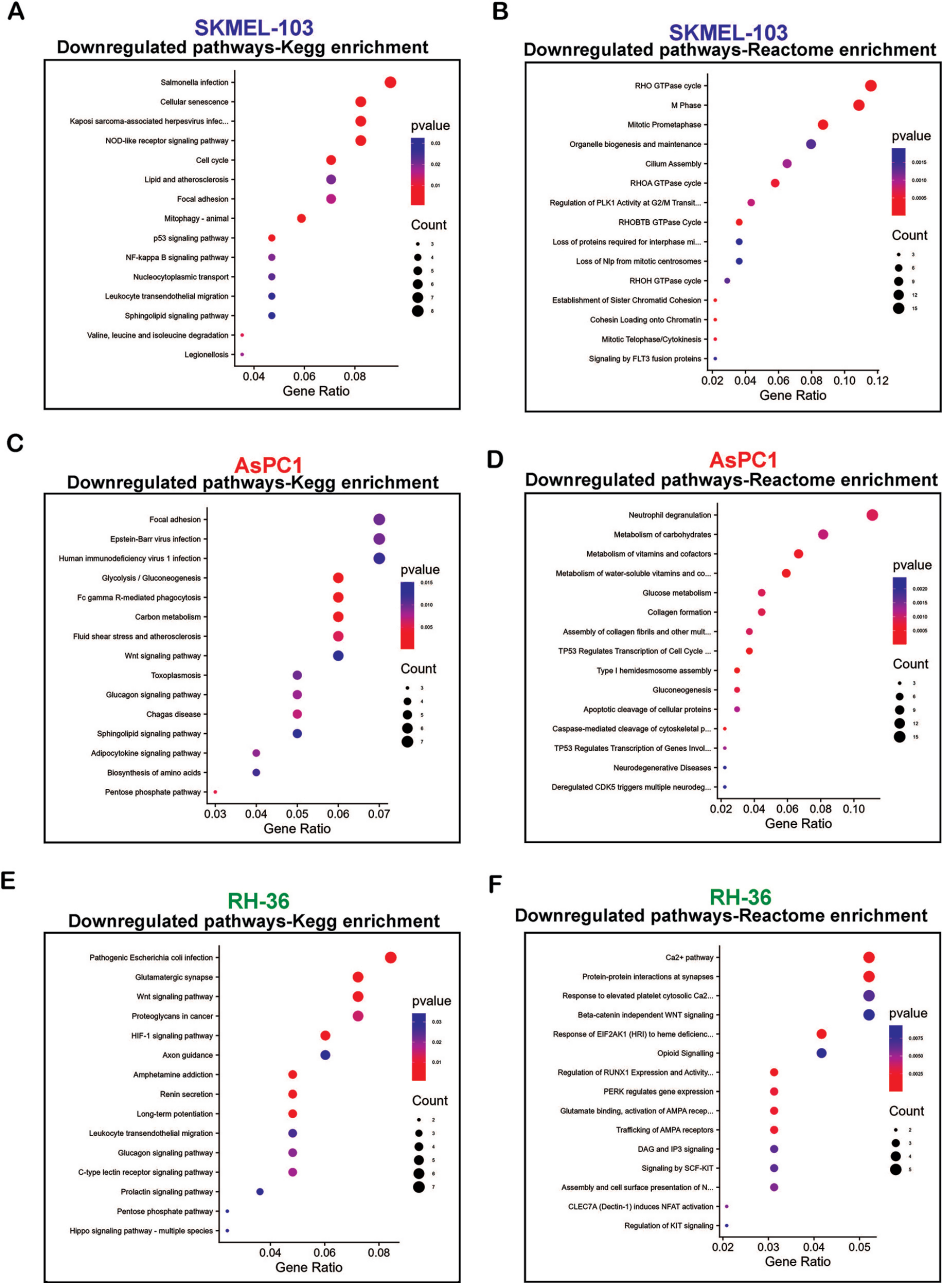
BAY 11-7082 treatment downregulates multiple pathways in NRAS, KRAS, and HRAS mutant cancer cells. **A, C, E.** Pathways analyzed via Kegg enrichment analysis that were significantly downregulated in SKMEL-103, AsPC1, and RH-36 cells upon treatment with BAY 11-7082 (5 µM) for 48 h as compared with DMSO-treated cells. **B, D, F.** Pathways analyzed via Reactome enrichment analysis that was significantly downregulated in SKMEL-103, AsPC1, and RH-36 cells upon treatment with BAY 11-7082 (5 µM) for 48 h as compared with DMSO-treated cells

We further conducted RNA sequencing analyses to identify novel genes and pathways altered by BAY 11-7082 treatment in context of NRAS, KRAS, and HRAS mutant cancer cells which leads to tumor growth inhibition. RNA sequencing analyses was performed on NRAS mutant SKMEL-103 cells, KRAS mutant AsPC1, and HRAS mutant RH-36 cells by treating them either with DMSO or BAY 11-7082. The analysis demonstrated changes in expression of several genes across NRAS, KRAS, and HRAS mutant cell lines following BAY 11-7082 treatment (Fig. 5, Supplementary Tables 1–3). Pathway enrichment analysis (Kegg enrichment and Reactome enrichment) performed using upregulated and downregulated genes identified several distinct functional pathways to be altered in response to BAY 11-7082 treatment (Fig. 6 and Supplementary Fig. 3) in NRAS, KRAS, and HRAS mutant cell lines. For example, in the KRAS mutant AsPC1 cell line, Kegg enrichment analysis revealed that BAY 11-7082 notably downregulated key metabolic pathways, including glycolysis, carbon metabolism, amino acid biosynthesis, and the pentose phosphate pathway. It also indicated that Wnt, glucagon, and sphingolipid signaling pathways were also suppressed. Furthermore, Reactome enrichment analysis revealed the same, namely BAY 11-7082 treatment downregulated several metabolic pathways, including carbohydrate metabolism, vitamins, cofactors, and gluconeogenesis (Fig. 6). These results indicated that BAY 11-7082 treatment majorly effects metabolic pathways in KRAS mutant cancer cells. These metabolic pathways have shown to plays a key role in cancer growth and progression and therefore their inhibition by BAY 11-7082 treatment may contribute to suppression of KRAS mutant cancer cells growth as observed in Figs. 1, 2 and 3.

In NRAS mutant cancer cells, Kegg enrichment analysis identified several downregulated pathways, which included nucleotide oligomerization domain (NOD)-like receptor signaling pathways, p53 signaling pathway, NF-kappa B signaling pathway, sphingolipid pathways among others. These pathway has shown to play key role in regulating several aspects of cancer growth. On the other hand, Reactome enrichment analysis identified RHO GTPase associated signaling pathway to be the main pathway downregulated upon BAY 11-7082 treatment in NRAS mutant cancer cell (Fig. 6). Rho-family GTPases have been shown to play an important role in cancer proliferation and migration [25]. Deregulation of these pathways may contribute to tumor growth inhibition of NRAS mutant melanoma cells upon BAY 11-7082 treatment as observed in Figs. 1, 2 and 3 of our result section.

Regarding HRAS mutant cancer cells, the downregulated pathways via Kegg enrichment analysis included Wnt signaling, hypoxia-inducible factor 1 (HIF-1) signaling, glucagon signaling, Prolactin signaling, and Hippo signaling, among others. Reactome enrichment analysis identified Protein kinase RNA- like endoplasmic reticulum kinase (PERK) signaling, diacylglycerol (DAG), and Inositol trisphosphate (IP3) signaling, KIT mediated signaling pathways as the important downregulated pathways in HRAS mutant cancer cells when they are treated with BAY 11-7082, (Fig. 6), which may contribute to tumor growth inhibition of HRAS mutant melanoma cells as observed in Figs. 1, 2 and 3 of our result section. Studies have shown that these pathways play an important role in tumor growth and targeting them has emerged as a promising strategy for cancer therapy.

In summary, all the pathways identified to be downregulated in NRAS, KRAS, and HRAS mutant cancer cell lines upon BAY 11-7082 treatment are prooncogenic and have shown to promote different aspects of tumor growth and progression. These results also indicate that although all RAS-mutant cancer cell lines display similar tumor growth inhibitory phenotypes (Figs. 1, 2 and 3) when treated with BAY 11-7082, however the mechanism underlying their tumor growth suppression is not identical and varies in context of different RAS. In conclusion, BAY 11-7082 can invariably be used for treating cancer cells expressing different RAS-mutants.

## Discussion

RAS proteins are a family of GTPases that play a crucial role in cell signaling pathways involved in cell growth, differentiation, and survival [26]. Mutations in the RAS genes are the most common genetic alterations found in human cancers [27, 28]. The three main RAS genes mutated in cancer are HRAS, KRAS, and NRAS [29]. Constitutive active RAS stimulates several downstream signaling pathways, including the RAF-MEK-ERK (MAPK) and the PI3K-AKT-mTOR signals pathways, which promotes uncontrolled cell growth and proliferation in cancer cells even in the absence of external growth signals. RAS mutations are often associated with high aggressiveness in tumors, predict poor prognosis, and affect response to certain targeted therapies and chemotherapy [30, 31]. Thus, targeting RAS has become important for cancer therapy. Studies have shown that directly targeting RAS is difficult due to its structure and dynamic behavior [32]. Therefore, efforts are being made to target downstream effectors and signaling pathways activated by mutated RAS in various cancer types. This includes targeting components of MAP kinase pathways such as RAF, MEK, and ERK and components of PI3K-AKT signaling pathways [33]. These inhibitors are successfully used in clinical trials to treat several cancer types [33]. SOS1 inhibitors are also under investigation for treating RAS-mutated cancers [33]. The biggest challenge in developing effective RAS-targeted therapies includes the development of resistance to these therapies, tumor heterogeneity [34], and the need for patient stratification based on RAS mutation types.

The current scenario demands exploring novel strategies for developing RAS-targeted therapies. In this regard, the development of inhibitors targeting all RAS protein family represents a significant area of focus. Some approaches and potential avenues in developing these inhibitors include developing covalent inhibitors that function by forming a robust and irreversible bond with the target protein. These allosteric inhibitors bind to the alternative sites and target downstream pathways that RAS activates [35]. A few covalent inhibitors that have shown promise in inhibiting KRAS G12C mutations, include AMG 510 (Sotorasib) and MRTX849 (Adagrasib) [33].

Identifying an inhibitor targeting all RAS protein family members would represent a significant stride forward in cancer therapeutics. It will provide a versatile solution to the genetic heterogeneity observed in different cancers harboring HRAS, NRAS, and KRAS, making it a valuable tool to treat various malignancies with RAS mutations. Inhibitors targeting all RAS protein family are emerging successfully as few of them have entered clinical trials for treating advanced solid tumors with HRAS, KRAS, and NRAS mutations (NCT06096974) in combination with trametinib (NCT05907304) [35].

**Fig. 7:**
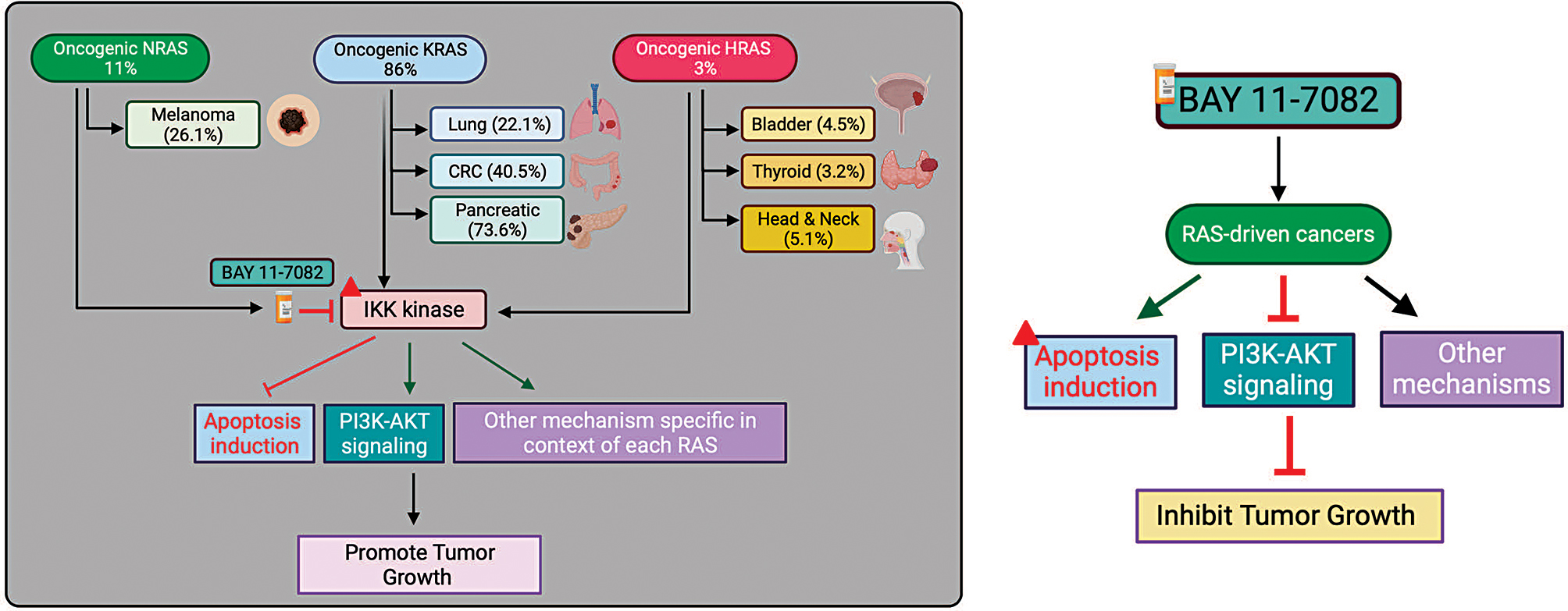
Model showing the new IκBα kinase Inhibitor BAY 11-7082 for Treating RAS-driven cancers. A model depicting various cancer types driven by oncogenic NRAS, KRAS, and HRAS mutations overexpress IκBα kinase. IκBα kinase can be effectively targeted by small molecule inhibitor BAY 11-7082, leading to downregulation of several prooncogenic signaling pathways, including PI3K-AKT signaling cascade and upregulation of apoptosis that contributes to tumor growth inhibition observed in RAS-driven cancers

Our study is summarized in Fig. 7. We find that BAY 11-7082, an inhibitor IκBα, effectively suppresses the growth of all RAS-mutant cancers. IκBα is a crucial regulatory protein that controls the nuclear factor kappa B (NF-κB) signaling pathway, acting as its suppressor [36, 37]. The NF-κB pathway plays a central role in regulating immune and inflammatory responses and cell survival, proliferation, and differentiation [38]. The relationship between IκBα and cancer is complex and context-dependent [39]. While IκBα is generally considered a negative regulator of the NF-κB pathway and, in that sense, has tumor-suppressive properties, there are scenarios in which IκBα may exhibit activities that could contribute to cancer progression [40, 41]. Additionally, depending on the cellular context, the NF-κB pathway can have both tumor-promoting and tumor-suppressive functions [42]. In our study, we find IκBα contributes to RAS-mediated tumorigenesis.

The BAY 11-7082 treatment exerts a broad-spectrum impact, mechanistically altering various pathways in cancer cells harboring NRAS, KRAS, and HRAS mutations. Specifically, this compound significantly downregulated the metabolic pathways in KRAS mutant cancer cells. Since metabolic reprogramming is a hallmark of cancer, supporting growth and proliferation [43] several inhibitors were evaluated in preclinical models and clinical trials targeting metabolic vulnerability in cancer cells, showing promising results [44, 45].

In NRAS mutant cancer cells, NOD-like receptor signaling and RHO GTPase-associated pathways were downregulated upon BAY 11-7082 treatment. Cytosolic NOD-like receptors are shown to regulate NF-kappa B signaling and MAPK pathway, which are important for tumor growth [46]. On the other hand, RHO GTPase-associated pathways have been shown to play a key role as signal transducers and participate in cell polarity, migration, and proliferation. Hence, its targeting represents a most promising approach to treat cancer [25, 47, 48]. RHO GTPase effector kinases-PAK and ROCK inhibitors are now tested in phase I clinical trials [49].

Regarding HRAS mutant cancer cells, the downregulated pathways included Wnt signaling, HIF-1 signaling, glucagon signaling, Prolactin signaling, and Hippo signaling. Wnt signaling plays an important role in regulating development and stemness in cancer [50]. HIF-1 signaling activates the transcription of crucial genes involved in tumor growth [51]. Prolactin signaling via Janus kinase-signal transducer and activator of transcription (JAK-STAT) is shown to promote tumor growth [52] and the Hippo pathway regulates metabolic reprogramming to promote the growth of malignant tumors [53]. These results highlight that many important pathways downregulated by BAY 11-7082 treatment impair the development of NRAS, KRAS, and HRAS mutant cancer. Accordingly, BAY 11-7082 could be considered an effective inhibitor targeting NRAS, KRAS, and HRAS mutant cancer and serve as a promising candidate for more effective treatments tailored to all RAS-mutant cancers. In conclusion, our study unravels a novel therapeutic approach and lays the foundation for advancing the landscape of RAS-mutant cancer treatments.

### Limitations of the study

Although, our study remains valuable for early screening and identifying BAY 11-7082 as a promising candidate for treating RAS-driven cancers, further in depth-investigation needs to be performed to gain comprehensive understating of the BAY 11-7082 effectiveness and safety using clinically relevant in vivo animal models and human clinical trials.

## Materials and methods

### Cell culture

RAS mutant cancer cell lines (NRAS: M245, SKMEL-103, SKMEL-2; KRAS: PANC1, AsPC1, SU.86.86) were purchased from American Type Culture Collection (ATCC) as listed in Supplementary Tables 4 and maintained in a humidified atmosphere of 5% CO2 at 37 °C in Dulbecco’s modified Eagle medium (Life Technologies, Carlsbad, CA, USA) or Roswell Park Memorial Institute-1640 Medium (Life Technologies), each supplemented with 10% fetal bovine serum and 1% penicillin/streptomycin (both from Life Technologies). HRAS mutant cell lines SMS-CTR, RH-36 cell lines were obtained from Christine A. Pratilas laboratory from Johns Hopkins University School of Medicine and grown as recommended.

### Chemical inhibitors

BAY 11-7082 (Cat. No.: HY-13,453) was purchased from Sigma and MedChemExpress and dissolved for cell culture and for in vivo experiments as suggested in the data sheet. Relevant information is provided in Supplementary Table 4.

### MTT (4,5-dimethylthiazol-2-yl)-2,5-diphenyltetrazolium bromide) assay

For MTT assay, 2 × 10^3^ of NRAS, KRAS and HRAS mutant cells were plated in a 100 µl volume in 96-well plates. After 24 h, BAY 11-7082 inhibitor, used at a range of concentrations (0.5µM, 1µM, 2µM, 5µM) was mixed in 100 µl of medium and added to the cells. After 3 days of inhibitor treatment, the cell viability was evaluated. To do this, 20 µl of 5 mg/ml MTT (1-(4,5-Dimethylthiazol-2-yl)-3,5-diphenylformazan) solution dissolved in 1× PBS was added to each well and incubated for 2 h at 37 °C incubator. The MTT solution was removed gently, and 100 µl of dimethyl sulfoxide (DMSO) were added. After mixing well by pipetting, absorbance was measured at 590 nm and 630 nm. An average was calculated for both readings, and then measurement at 630 nm was subtracted from that at 590 nm. The relative cell viability was plotted with respect to control DMSO treated cells.

### Clonogenic assay

For clonogenic assay, NRAS mutant cells (M245 (1 × 10^3^), SKMEL-103 (1 × 10^3^), SKMEL-2 (2 × 10^3^)), KRAS mutant cells (PANC1 (1.5 × 10^3^), AsPC1 (1.5 × 10^3^), SU.86.86 (1.5 × 10^3^)) and HRAS mutant cells (SMS-CTR (1 × 10^3^), RH-36 (1 × 10^3^)) were seeded in a six-well plate. Cells were seeded in triplicate in 6-well plate and after 24 h they were either treated with DMSO or with different concentrations of BAY 11-7082. After 3–4 weeks, colonies formed were fixed using a fixing solution containing 50% methanol and 10% acetic acid and then stained with 0.05% coomassie blue (Sigma-Aldrich, St. Louis, MO, USA). Representative images each sample under the indicated conditions is shown.

### Soft-agar assay

Soft-agar assays were performed by seeding NRAS mutant cells (M245 (2 × 10^3^), SKMEL-103 (2 × 10^3^), SKMEL-2 (2 × 10^3^)), KRAS mutant cells (PANC1 (2 × 10^3^), AsPC1 (2 × 10^3^), SU.86.86 (2 × 10^3^)) and HRAS mutant cells (SMS-CTR (2 × 10^3^), RH-36 (2 × 10^3^)) onto 0.4% low-melting-point agarose (Sigma-Aldrich) layered on top of 0.8% agarose. After 24 h, they were either treated with DMSO or with different concentrations of BAY 11-7082. After 3–6 weeks of incubation, colonies formed were stained with a 0.05% crystal violet solution and imaged using a microscope. Colony size was measured using microscopy and ImageJ software (https://imagej.nih.gov/ij/) and plotted as the percent relative colony size compared with control DMSO treated cells. Statistical analysis was performed using the two-tailed unpaired Student’s *t*-test in GraphPad Prism software, version 7.0, for Macintosh.

### Immunoblotting analysis

Whole-cell protein extracts were prepared using RIPA lysis buffer (Pierce) containing Protease Inhibitor Cocktail (Roche) and Phosphatase Inhibitor Cocktail (Sigma-Aldrich, St. Louis, MO). Lysed samples were centrifuged at 12,000 rpm for 40 min, and clarified supernatants were stored at − 80 °C. Protein concentrations were determined using Bradford Protein Assay Reagent (Bio-Rad Laboratories, Hercules, CA, USA). Equal amounts of protein samples were electrophoresed on 10% or 12% sodium dodecyl sulfate (SDS)- polyacrylamide gels and transferred onto polyvinylidene difluoride (PVDF) membranes (Millipore, Burlington, MA, USA) using a wet-transfer apparatus from Bio-Rad. The membranes were blocked with 5% skim milk and probed with primary antibodies in 5% BSA. After washing, the membranes were incubated with the appropriate horseradish peroxidase (HRP)-conjugated secondary antibodies (1:2,000) (GE Healthcare Life Sciences, Marlborough, MA, USA). The blots were developed using SuperSignal West Pico or Femto Chemiluminescent Substrate (Thermo Fisher Scientific). All antibodies used for immunoblotting are listed in Supplementary Table 4.

### Apoptosis measurement using annexin V/propidium iodide staining

Annexin V binding to cells was measured with the use of an Annexin V staining kit (BD PharmingenTM #556,547, BD Pharmingen, San Diego, CA, USA) according to the manufacture’s protocol. In brief RAS mutant cells were treated with vehicle or inhibitor (5 μm) for 48 h. After treatment, cells were collected, washed twice with 1× PBS and resuspended in 1× Binding buffer and stained with 5 µL FITC-Annexin V and 5 µL of PI and incubated for 15 min in the dark. After incubation, cells were analyzed with FACS using LSR Fortessa (BD Biosciences, Franklin Lakes, NJ, USA).

### Subcutaneous xenograft-based mouse tumorigenesis experiment with BAY 11-7082 treatment

NRAS; SKMEL-103 (2 × 10^6^), KRAS; AsPC1 (5 × 10^6^) cells and HRAS; RH-36 (7 × 10^6^),in 100 µl mixed with 100 µl of matrigel were injected subcutaneously into 5–6-week-old NSG mice (stock No. 005557). Tumor volume was measured every week, and tumor size was calculated using the following formula: length × width^2^ × 0.5. When the tumor volumes reached ∼80–100 mm^3^, the mice were treated with either vehicle (0.5% methyl cellulose in water) or BAY 11-7082 (15 mg/kg body weight) intraperitoneally every other day until the end of the experimental period. Tumor volume was measured every week and plotted. Subcutaneous tumors from individual groups were harvested and imaged. All protocols for mouse experiments were approved by the Institutional Animal Care and Use Committee of the University of Alabama at Birmingham (UAB). Additionally, subcutaneous tumors obtained from vehicle and BAY 11-7082 treated condition were sectioned and stained for H&E and Ki-67. For H&E staining, tumor tissues are washed with cold PBS and fixed in 4% formalin. These tumors are then sectioned at 5 μm thickness, embedded in paraffin and the sections are then processed for histological analysis. First the section are washed, deparaffinized, dehydrated, and stained with H&E. For Ki-67 staining, tumor tissues containing slides were stained for standard IHC with anti-Ki-67 antibody (1:200). Briefly, following slide deparaffinization, antigen retrieval was performed in citrate buffer (pH 6.0) at 97 °C for 20 min, using the Lab Vision PT Module (ThermoFisher Scientific). Endogenous peroxides were blocked by incubation in hydrogen peroxide for 30 min, followed by washing with 1× Tris-buffered saline, and proteins were blocked by incubation with 0.3% BSA for 30 min. Slides were incubated in anti-Ki67 antibody (dilution 1:200) followed by secondary anti-rabbit HRP-conjugated antibody (Dako, Jena, Germany). Three randomly chosen magnification fields were imaged from each tumor (vehicle and BAY 11-7082 treated). All antibodies used for immunohistochemistry analyses are listed in Supplementary Table 4.

### RNA sequencing and data analysis

SKMEL-103, AsPC1 and RH-36 cells were treated with DMSO (control) or BAY 11-7082 (5 µM) for 48 h. Total RNA was extracted using TRIzol® reagent (Invitrogen, Carlsbad, CA, USA) according to the manufacturer’s instructions and purified on RNAeasy mini columns (Qiagen, Hilden, Germany) according to the manufacturer’s instructions. Then, mRNA was purified from approximately 500 ng total RNA using oligo-dT beads and sheared by incubation at 94 °C. Following first-strand synthesis with random primers, second-strand synthesis was performed with dUTP to generate strand-specific libraries. The cDNA libraries were then end-repaired and A-tailed. Adapters were ligated, and second-strand digestion was performed using uracil-DNA-glycosylase. Indexed libraries that met appropriate cutoffs for both were measured by quantitative reverse transcription polymerase chain reaction (qRT-PCR) using a commercially available kit (KAPA Biosystems, Wilmington, MA, USA). The insert-size distribution was determined using LabChip GX (PerkinElmer, Waltham, MA, USA) or an Agilent Bioanalyzer (Agilent Technologies, Santa Clara, CA, USA). Samples with a yield ≥ 0.5 ng/µL were used for sequencing on the Illumina HiSeq 2500 system (Illumina, San Diego, CA, USA). Images were converted into nucleotide sequences by the base-calling pipeline RTA 1.18.64.0 and stored in FASTQ format. For data analysis, the reads were first mapped to the latest UCSC transcript set using Bowtie2 version 2.1.0, and the gene expression level was estimated using RSEM v1.2.15. The Trimmed Mean of the M-values method was used to normalize the raw count. Differentially expressed genes were identified using the edgeR program. Genes showing altered expression with *p* < 0.05 and more than 1.5-fold changes were considered differentially expressed. Clusterprofiler was used for the Gene Ontology and pathway enrichment analyses. RNA-sequencing data presented in this paper are submitted to Gene Expression Omnibus (Accession No. GSE251968) and available publicly without restrictions.

### Statistical analysis

All experiments were conducted with at least three biological replicates. Results for individual experiments are expressed as mean ± standard error of the mean (SEM). For the analysis of tumor progression in mice, the statistical assessment was performed using using two-way anova on GraphPad Prism version 10.2.1 for Macintosh (GraphPad Software, LLC). The *p*-values for the rest of the experiments were calculated using the two-tailed unpaired Student’s *t*-test in GraphPad Prism software, version 7.0, for Macintosh.

### Electronic supplementary material

Below is the link to the electronic supplementary material.

Supplementary Material 1

Supplementary Material 2

Supplementary Material 3

Supplementary Material 4

Supplementary Material 5

Supplementary Material 6

Supplementary Material 7

## Data Availability

RNA-sequencing data presented in this paper are submitted to Gene Expression Omnibus (Accession No. GSE251968) and available publicly without restrictions. Additionally, any information presented in the current study are available from the corresponding author on reasonable request.
