# Supplementary material for: IκBα kinase inhibitor BAY 11-7082 promotes anti-tumor effect in RAS-driven cancers
Source: J Transl Med. 2024 Jul 9;22:642. doi: 10.1186/s12967-024-05384-4 (PMC11233160; doi:10.1186/s12967-024-05384-4)
Supplement: Supplementary file 4 — Supplementary Material 4 [file 12967_2024_5384_MOESM4_ESM.pdf]

Supplemental information

Supplementary Figures

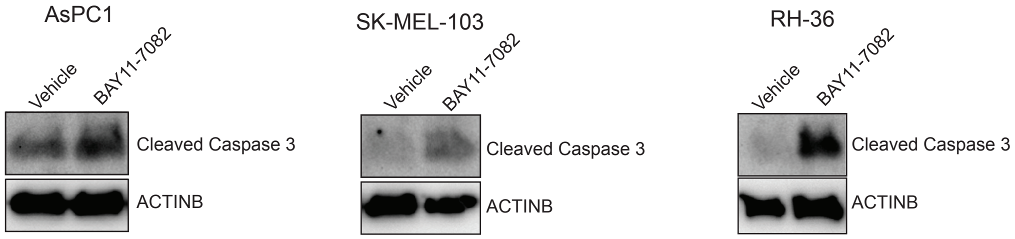

Supplementary Figure 1. BAY 11-7082 treatment *in vivo* upregulates apoptosis. Lysate was prepared from the tumors obtained from vehicle or BAY 11-7082 treated condition and cleaved caspase 3 level was were measured via immunoblotting. ACTINB was used as loading control.

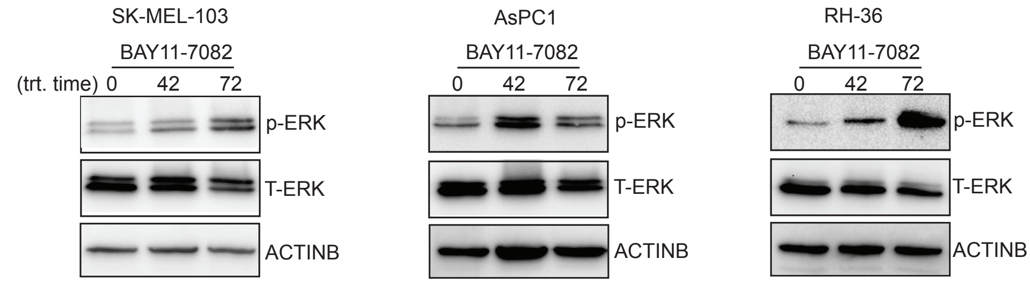

Supplementary Figure 2. Effect of BAY 11-7082 treatment on MAPK signaling. The indicated cancer cell lines were treated with BAY 11-7082 (5 μM) for 48 and 72 hours, and phosphorylated ERK and total ERK were measured via immunoblotting. ACTINB was used as loading control.

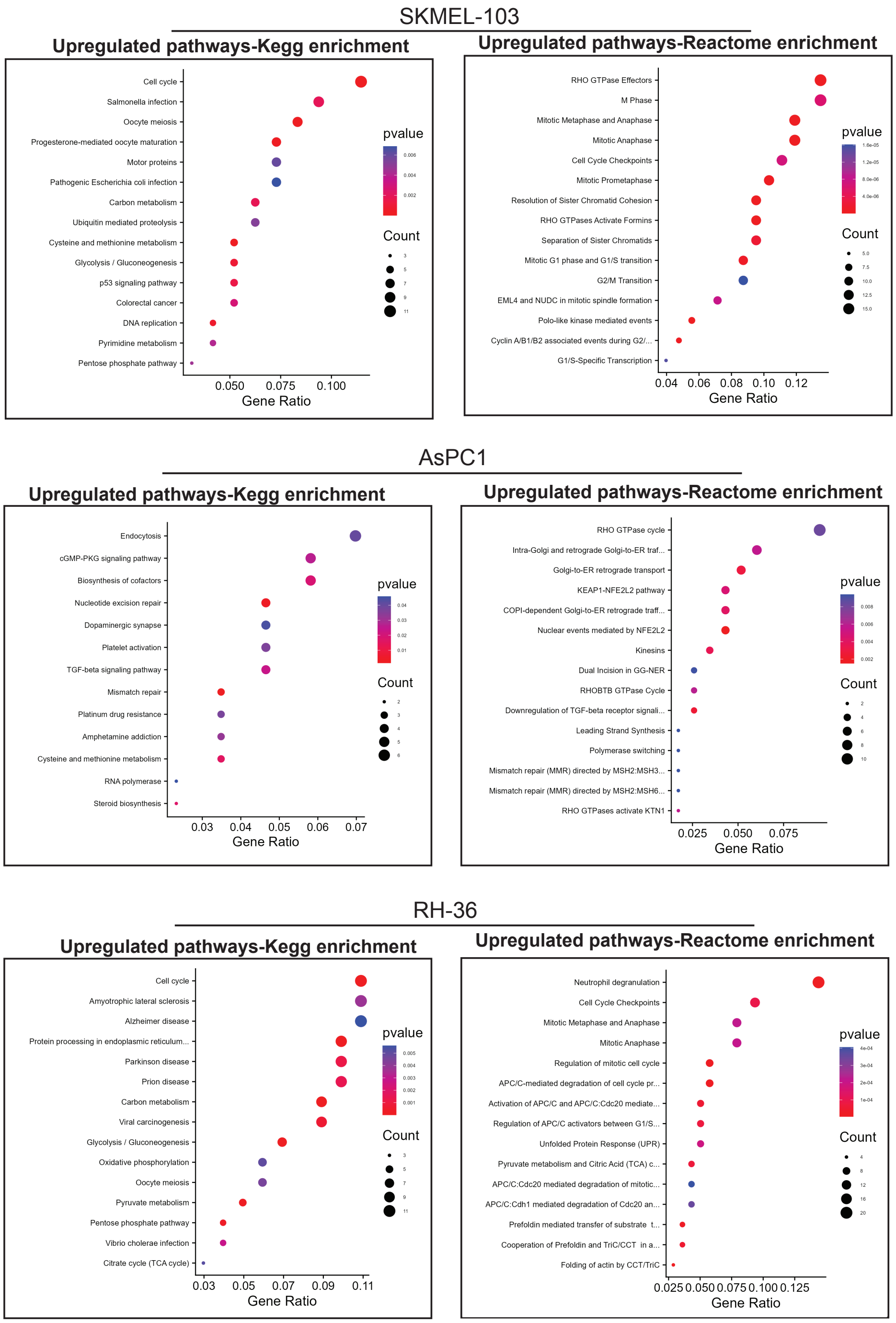

Supplementary Figure 3. BAY 11-7082 treatment upregulates multiple specific pathways in NRAS, KRAS, and HRAS mutant cancer cells. A, C, E. Pathways analyzed via Kegg enrichment analysis that was significantly upregulated in SKMEL-103, AsPC1, and RH-36 cells treated with BAY 11-7082 (5 µM) for 48 h compared with DMSO-treated cells. **B, D, F.** Pathways

analyzed via Reactome enrichment analysis that was significantly upregulated in SKMEL-103, AsPC1, and RH-36 cells treated with BAY 11-7082 (5 μM) for 48 h compared with DMSO-treated cells.

Supplementary Tables:

Supplementary Table 1: List of differentially regulated genes identified from RNA-sequencing analysis of SKMEL-103 cells after 48-h treatment with BAY 11-7082 (5 μM) or control DMSO.

Supplementary Table 2: List of differentially regulated genes identified from RNA-sequencing analysis of AsPC1 cells after 48-h treatment with BAY 11-7082 (5 μM) or control DMSO.

Supplementary Table 3: List of differentially regulated genes identified from RNA-sequencing analysis of RH-36 cells after 48-h treatment with BAY 11-7082 (5 μM) or control DMSO.

Supplementary Table 4: List of Reagents, data and software used in this study with source and identifier.
