# Supplementary material for: IκBα kinase inhibitor BAY 11-7082 promotes anti-tumor effect in RAS-driven cancers
Source: J Transl Med. 2024 Jul 9;22:642. doi: 10.1186/s12967-024-05384-4 (PMC11233160; doi:10.1186/s12967-024-05384-4)

Raw Supplemental Data 1

Raw blots for Figure 4A
SKMEL-103

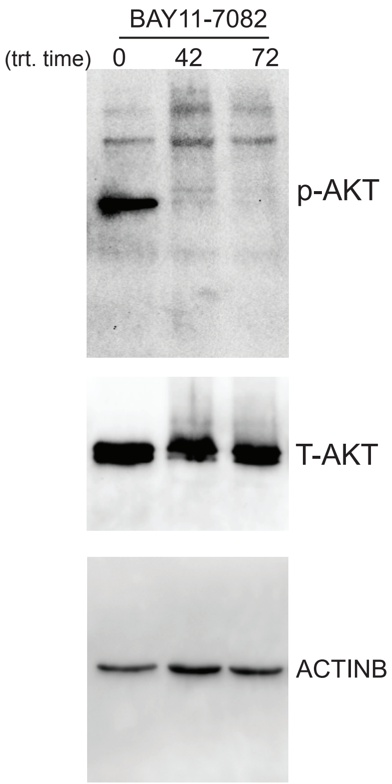

Raw blots for Figure 4E
AsPC1

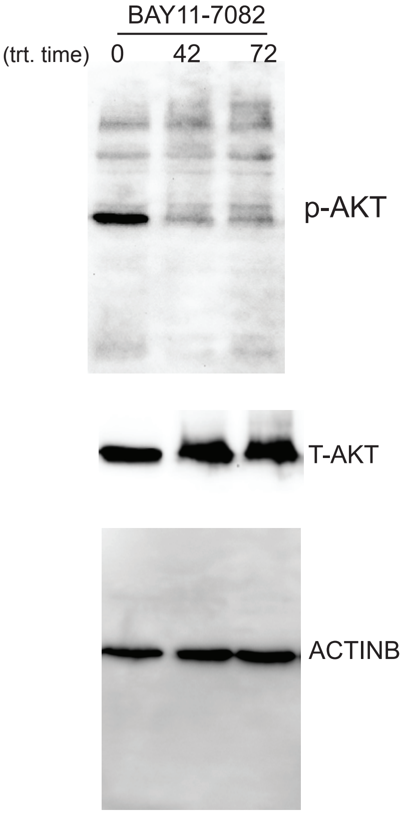

Raw blots for Figure 4I
RH-36

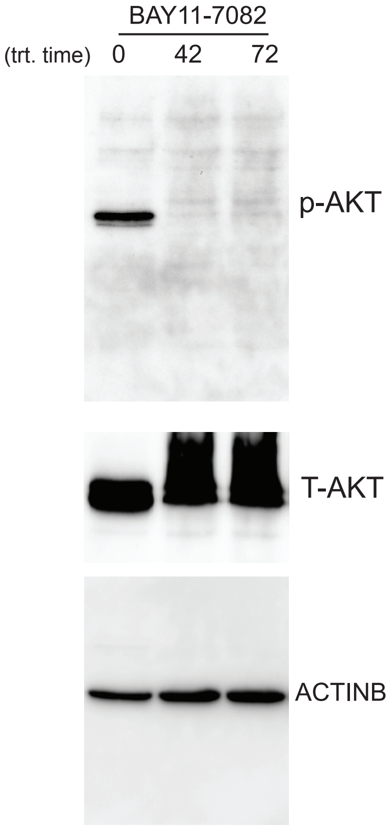

Raw blots for Figure 4B
SKMEL-103

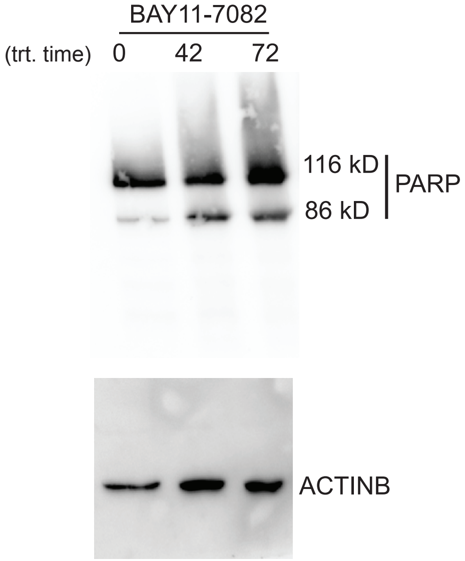

Raw blots for Figure 4F
AsPC1

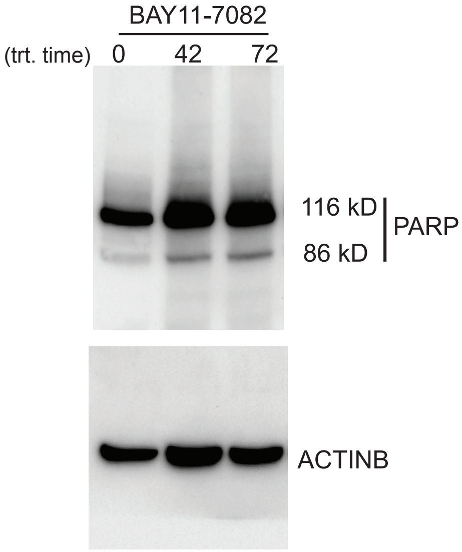

Raw blots for Figure 4J
RH-36

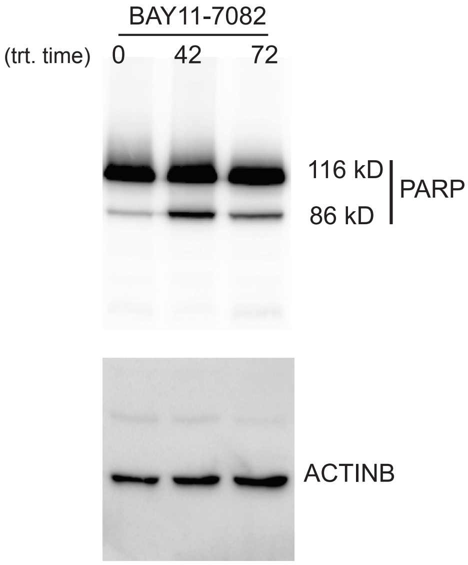

Raw Supplemental Data 2

Raw blots for Supplementary Figure 1

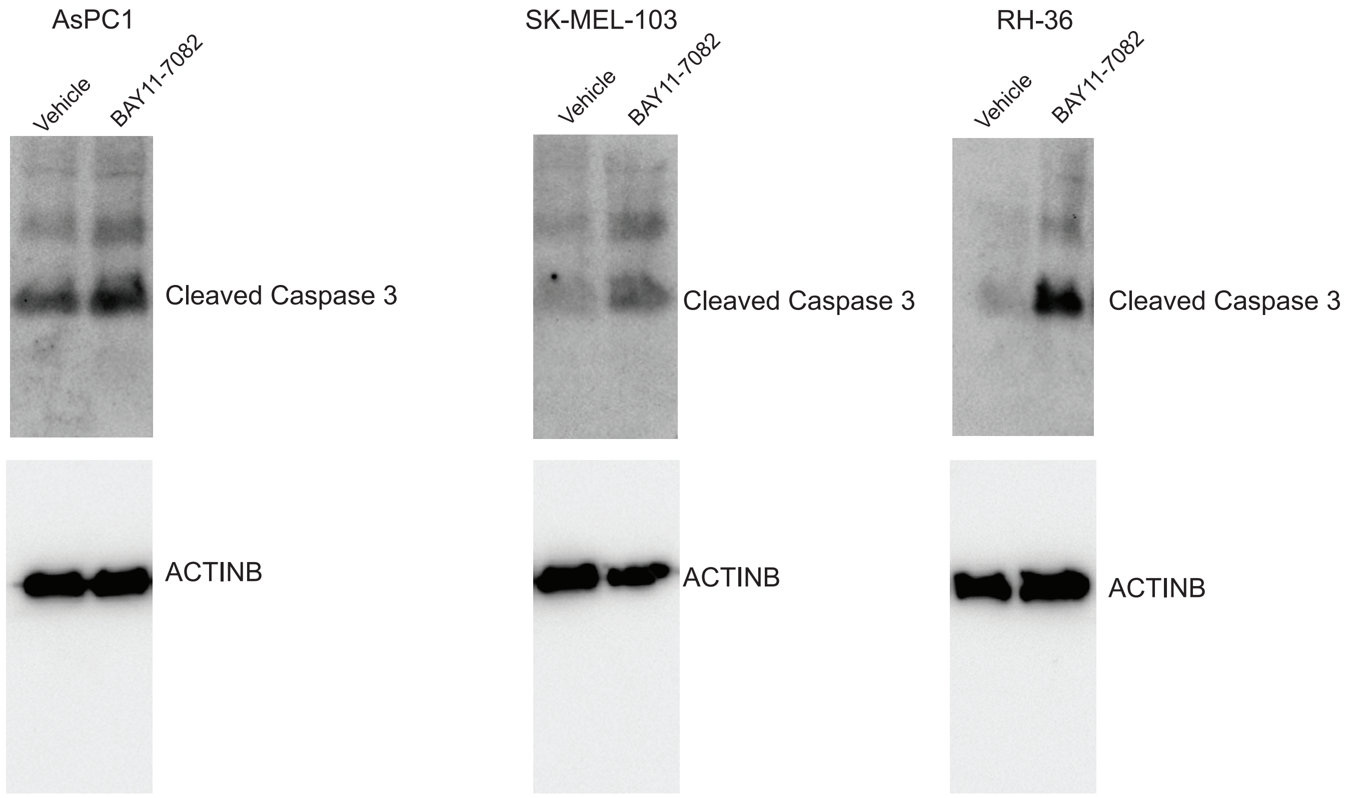

Raw blots for Supplementary Figure 2

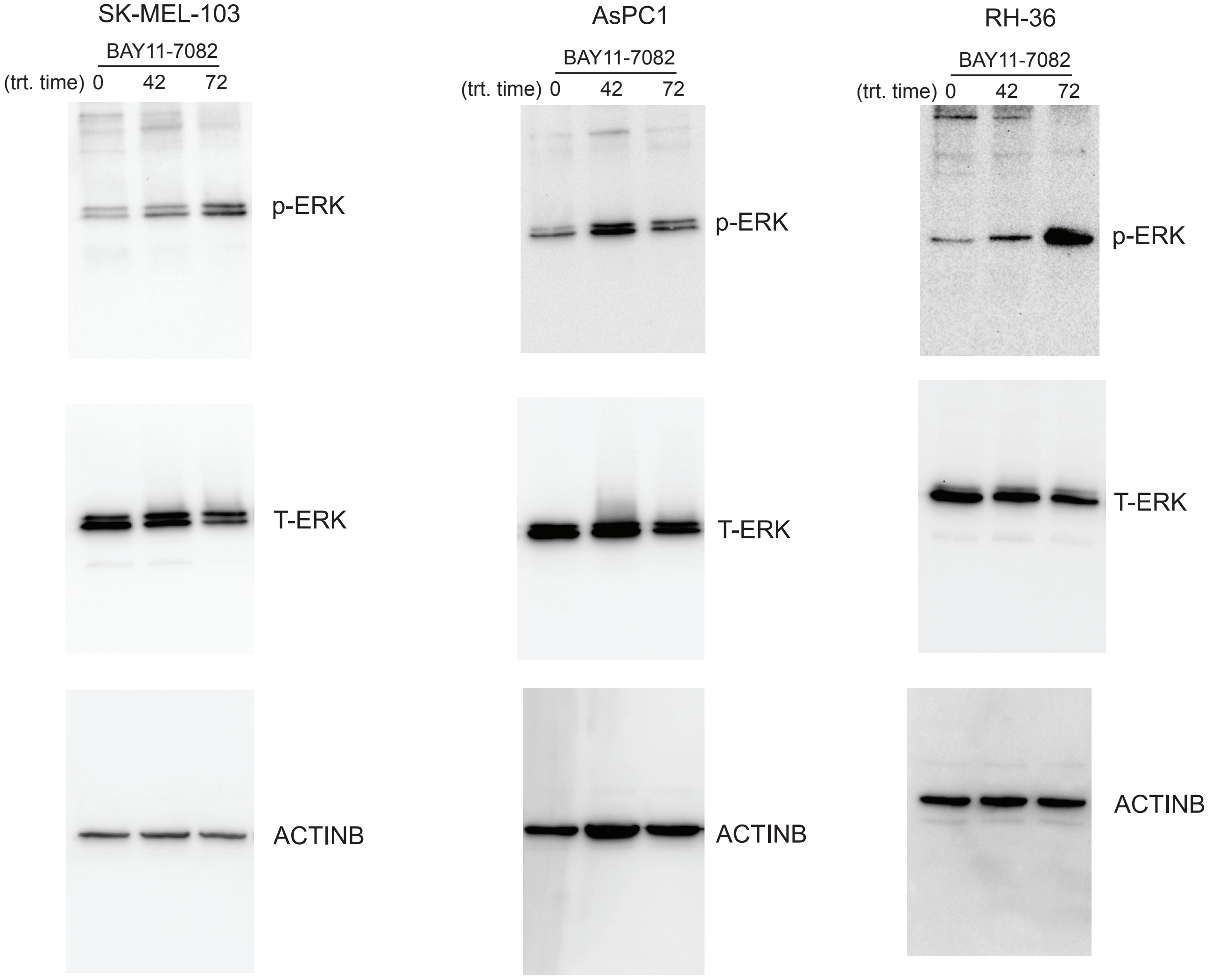

Supplement: Supplementary file 5 — Supplementary Material 5 [file 12967_2024_5384_MOESM5_ESM.pdf]
