# Supplementary material for: IκBα kinase inhibitor BAY 11-7082 promotes anti-tumor effect in RAS-driven cancers
Source: J Transl Med. 2024 Jul 9;22:642. doi: 10.1186/s12967-024-05384-4 (PMC11233160; doi:10.1186/s12967-024-05384-4)
Supplement: Supplementary file 6 — Supplementary Material 6 [file 12967_2024_5384_MOESM6_ESM.pdf]

Supplementary Table 4

Key Resource Table. List of reagents, data, and software used in this study with source and identifier.

**Reagents**		
**Inhibitor and**	**Concentrations**	**Source**
BAY 11-7082	Different concentration	Sigma,MedChem Express
**Antibodies**	**Source**	**Identifier**
PARP	Cell Signaling	Cat# 9542
ACTINB	Cell Signaling	Cat#4970
phospho-AKT	Cell Signaling	Cat# 9271S
Total AKT	Cell Signaling	Cat# 9272S
Phospho-ERK	Cell Signaling	Cat# 4376S
Total ERK	Cell Signaling	Cat# 4695S
Ki-67	Thermo Fisher Scientific	Cat# RM9106S0
**Commercial assays and kits**		
FITC-Annexin V Apoptosis Detection Kit I	BD Pharmingen	Cat# 556547
**Software and Algorithms**		
Prism 9.0	Prism 9.0	Prism 9.0
ImageJ	https://imagej.nih.gov/ij	N/A
**Deposited Data**		
RNA-Seq performed with SKMEL-103, AsPC1 and RH-36 cells treated with BAY 11-7082 or DMSO	This paper	GSE251968
**Experimental Models: Organisms/Strains**		
Mouse: NSG	Jackson Laboratory	Stock No. 005557
**Chemicals, Peptides, and Recombinant Proteins**		
DMEM	Sigma-Aldrich	Cat# D5796
RPMI	Sigma-Aldrich	Cat# R8758
Fetal Bovine Serum	GIBCO	Cat# 10437-028
Trypsin-EDTA	GIBCO	Cat# 25200-056
Penicillin-Streptomycin	GIBCO	Cat# 15140-122
Effectene Transfection Reagent	QIAGEN	Cat# 301427
Agarose, Low gelling	Sigma-Aldrich	Cat# A9045
