# Supplementary material for: IκBα kinase inhibitor BAY 11-7082 promotes anti-tumor effect in RAS-driven cancers
Source: J Transl Med. 2024 Jul 9;22:642. doi: 10.1186/s12967-024-05384-4 (PMC11233160; doi:10.1186/s12967-024-05384-4)
Supplement: Supplementary file 7 — Supplementary Material 7 [file 12967_2024_5384_MOESM7_ESM.pdf]

THE UNIVERSITY OF
ALABAMA AT BIRMINGHAM

**Romi Gupta, Ph.D.**
Assistant Professor
Department of Biochemistry and Molecular Genetics
Associate Scientist, Experimental Therapeutics Program, UAB Comprehensive Cancer Center
The University of Alabama at Birmingham
KAUL 540B, 720 20th Street South, Birmingham, AL 35233
P: 205.934.6207: romigup@uab.edu

June 3rd, 2023

Francesco Marincola, MD,
Editor-in-Chief
*Journal of Translational Medicine*

Dear Editor,

Thank you for your email dated May 7th and for forwarding the comments from the two reviewers. The reviewers acknowledged many strengths of our manuscript and noted that our study presents interesting data about the antitumor effects of BAY 11-7082 in RAS-driven cancers. Both Reviewer 1 and Reviewer 2 raised a few issues suggesting revisions to the manuscript. The suggestions from both reviewers were constructive and have helped us to further strengthen the manuscript. Please find attached a revised version of our manuscript, modified in light of the reviewers' suggestions. As you will see, we have fully addressed the suggestions from both the reviewers. Consequently, all the concerns of both the reviewers have now been addressed in this revised manuscript.

Our detailed point-by-point responses to the reviewers' comments are appended below. To facilitate the review of this revised manuscript, all changes made to address the reviewers' comments are highlighted in blue font in this marked-up manuscript. We hope that you find the revised manuscript now suitable for publication in the *Journal of Translational Medicine*. Please let me know if I can provide any additional information. Thank you very much for overseeing our manuscript.

Sincerely,

Romi Gupta

Romi Gupta

**Response to reviewer's comments:**

**Reviewer #1:** *The topic sounds interesting, and the results are reliable. It is worth to be published. However, the manuscript was not well organized, and there still has some unclear points and questions.*

We thank the reviewer for his/her supportive comments. Reviewer mentioned that "The topic sounds interesting, and the results are reliable. It is worth to be published*"*. However, the reviewer raised few issues. Our responses to reviewer's questions are appended below.

*Please define all abbreviations at their first appearance.*

We thank reviewer for this suggestion. As per reviewer's recommendation we have defined all the abbreviations at the first appearance throughout the manuscript.

*Page 4. Authors described "This analysis revealed that oncogenic forms of NRAS, KRAS, and HRAS activate the expression of IkappaBalpha (IκBα) kinase. This could not be confirmed in the references mentioned above. Please be more thorough and detailed as this will form the basis of this paper.*

We apologize that this information was not provided in detail. We now provide details of our previous paper, which formed the basis of our current study. Please see revised result section of the manuscript.

*Why did the authors target NRAS mutant- SKMEL-103 cell, KRAS mutant- AsPC1, and HRAS mutant-RH-36 cells in the study from Fig. 3 onwards?*

The cell lines chosen (NRAS mutant- SKMEL-103 cells, KRAS mutant- AsPC1 cells, and HRAS mutant-RH-36 cells) from Fig. 3 onwards were selected based on their ability to grow easily and robustly in culture, as well as in mice, thereby enabling us to obtain meaningful results in both cell culture and in *in vivo* experiments. Moreover, these cells have been used by others to study the oncogenic effects of mutant NRAS, mutant KRAS and mutant HRAS and thus, are well established prototypical models for such studies.

*Page 5. Please correct BAY 11-7082 (20 mg/kg) to BAY 11-7082 (15 mg/kg). Please look for any other errors?*

We have now corrected this information. We thank reviewer for noticing this.

*What is the effect on normal mice and surrounding normal tissues treated with BAY 11-7082?*

BAY11-7082 is specifically used to target IκBα kinase, whose expression is upregulated by oncogenic NRAS, KRAS, and HRAS in tumor cells. It is important to note that normal mice or cells or tissue do not naturally express IκBα kinase, and its expression and activity are finely tuned in response to intrinsic and extrinsic signaling. Therefore, administering BAY 11-7082 to normal mice would not align logically with its intended purpose. However, at the dose of 15 mg/kg of BAY 11-7082 that we used for our *in vivo* study, we did not note any observable toxicity during the course of drug treatment as it did not cause any enhanced mortality or any observable signs of sickness in mice. Based on these observations and the fact that RAS oncogene induced activation of IκBα kinase provides a therapeutic window to treat cancer cells selectively, BAY 11-7082 can be exploited for therapeutic purposes without significant toxicities.

*Please describe the methods of tumor collection, staining, and observation.*

As per reviewer's recommendation we have now provided detailed information on tumor collection, staining, and observation. Please refer to the revised method section.

*Page 6. Why did you use BAY 11-7082 at a concentration of 1 μM for apoptosis evaluation? This is the concentration at which no effect was seen in Fig.1 and Fig.2. In addition, I have some doubts about the gate settings of annexin V/PI. Why is the FITC gate shifted to the right only in RH-36? Furthermore, why is the positive rate of AsPC1 high even in the control group?*

We apologize for mislabeling the concentration as 1μM. The correct concentration is 5μM. We have now rectified this information in the revised manuscript. Additionally, we have adjusted the gate setting for the experiments. Please refer to the revised figure 4.

Furthermore, the high positive rate of AsPC1, even in the control group, could be attributed to its high proliferation rate, which might induce cell stress and trigger apoptosis. The ability of cancer cells to divide and baseline apoptotic index is an inherent cellular property that cannot be controlled or altered.

**Reviewer #2:**

*The manuscript of Guruvaiah and coauthors presents interesting data about the antitumor effects of BAY117082 in RAS driven cancers. Nonetheless, the article requires some major changes to be suitable for publication. A list of major and minor points that the authors should address is reported below.*

We thank reviewer for his/her encouraging words. Please see our response to reviewer's major and minor comments below.

Major comments:

*The paragraph of results should be detailed. In particular, authors should specify the results reported in figure 2A-E and in figure 5 and 6. Figure 2F is not cited and discussed.*

We thank reviewer for this important suggestion. We have now specified the results of Figure 2A-E in figure 5 and 6. We also apologize for not citing Figure 2F. It is now corrected in the revised manuscript.

*I suggest to use two-way anova to compare the curves in figure 3B.*

As recommended by reviewer, we have included two-way anova to compare the tumor growth presented in Figure 3. Please see revised figure 3B.

*The authors demonstrated the effect of BAY117082 on MAP kinase and PI3K-AKT signaling pathway but only pAKT and AKT were tested. Authors should also include pERK/ERK modulation. In figure 4A and 4E ACTINB is missing.*

As per the reviewer's recommendation, we have included the effect of BAY 11-7082 on the MAP kinase signaling pathway. Although we observed a significant decrease in the PI3K-AKT signaling pathway upon treatment with BAY 11-7082, we did not observe much change in the MAP kinase signaling pathway. Please refer to Supplementary figure 2 for details. Additionally, we have included ACTINB in figures 4A and 4E. Please see the revised figure 4 for the updated information.

*Authors demonstrated the pro-apoptotic effect of BAY117082 in cell lines. Histological examination of tumor tissues stained with a marker of apoptosis (for example cleaved caspase-3) should be provided in figure 3C.*

Based on reviewer's suggestion we measured cleaved caspase 3 from the tumor tissue obtained from vehicle and BAY 11-7082 treated mice. We observed increased cleaved caspase 3 levels in BAY 11-7082 treated mice as compared to vehicle treated mice. Please see Supplementary figure 1.

*Authors should specify why they used different doses of the drug and different times of treatment (48 and 72 h).*

Utilizing various doses of a drug and adjusting the duration of treatment in experiments offers several advantages, including identifying the optimal dose for achieving maximum tumor inhibitory effect. Additionally, it assists in assessing the efficacy of the drug across a range of concentrations, thereby enhancing the robustness of our study. In accordance with the reviewer's suggestion, we have now included this information in our manuscript. Please refer to the revised results section for details.

*Introduction resulted confused, I suggest you to rewrite it by including tumor types reported in figure 7.*

We have now revised the introduction section as per reviewer's suggestion. Please see revised introduction section.

*Please, add in figure 7 the effects of BAY117082 by including arrows showing activation of apoptosis and inhibition of PI3/AKT signaling pathway and other mechanisms. Please, move figure 7 in the discussion section.*

We have now added the effect of BAY 11-7082 on apoptosis induction and inhibition of PI3K/AKT signaling pathway in figure 7. Please see the revised figure. We have also moved figure 7 in the discussion section.

<u>Minor comments:</u>

*The dose used for BAY117082 is different in results (page 5) and in figure 3 legend.*

We thank reviewer for noticing this and have now corrected the dose.

*Please, modify the bar graphs with a scale of 100% (figure 1 A,B,C and 2 B,D,F).*

We would like to mention that if bar graphs are set with a scale of 100%, we will not be able to see the standard error for mean for many cases and the data will not be presented properly.

*Some language mistakes should be corrected.*

We have reviewed the manuscript entirely and have corrected the language as suggested by reviewer.
